# The Association between Cold Spells and Pediatric Outpatient Visits for Asthma in Shanghai, China

**DOI:** 10.1371/journal.pone.0042232

**Published:** 2012-07-25

**Authors:** Yuming Guo, Fan Jiang, Li Peng, Jun Zhang, Fuhai Geng, Jianming Xu, Canming Zhen, Xiaoming Shen, Shilu Tong

**Affiliations:** 1 School of Public Health and Institute of Health and Biomedical Innovation, Queensland University of Technology, Brisbane, Australia; 2 Department of Developmental and Behavioral Pediatrics, Shanghai Institute of Pediatric Translational Medicine, Shanghai Children's Medical Centre, Shanghai Jiaotong University, School of Medicine, Shanghai, China; 3 Ministry of Education-Shanghai Key Laboratory of Children's Environmental Health, Xinhua Hospital, Shanghai Jiaotong University, School of Medicine, Shanghai, China; 4 Shanghai Meteorological Bureau, Shanghai, China; Hospital for Sick Children, Canada

## Abstract

**Background:**

Asthma is a serious global health problem. However, few studies have investigated the relationship between cold spells and pediatric outpatient visits for asthma.

**Objective:**

To examine the association between cold spells and pediatric outpatient visits for asthma in Shanghai, China.

**Methods:**

We collected daily data on pediatric outpatient visits for asthma, mean temperature, relative humidity, and ozone from Shanghai between 1 January 2007 and 31 December 2009. We defined cold spells as four or more consecutive days with temperature below the 5^th^ percentile of temperature during 2007–2009. We used a Poisson regression model to examine the impact of temperature on pediatric outpatient visits for asthma in cold seasons during 2007 and 2009. We examined the effect of cold spells on asthma compared with non-cold spell days.

**Results:**

There was a significant relationship between cold temperatures and pediatric outpatient visits for asthma. The cold effects on children's asthma were observed at different lags. The lower the temperatures, the higher the risk for asthma attacks among children.

**Conclusion:**

Cold temperatures, particularly cold spells, significantly increase the risk of pediatric outpatient visits for asthma. The findings suggest that asthma children need to be better protected from cold effects in winter.

## Introduction

Asthma is one of the most important chronic diseases worldwide. It is estimated that there are about 300 million people with asthma currently in the world [1]. Asthma accounts for about one percent of all disability-adjusted life years lost worldwide, which reflects the severity and high prevalence of this disease [1]. The prevalence of asthma has been increasing in both children and adults around the world in recent decades [2], [3]. However, there is no treatment to cure it. Therefore, it is urgently required to identify the causes and/or risk factors for the onset of asthma so that effective control and prevention strategies can be developed.

There is sufficient evidence suggesting that air pollution (e.g., ozone) decreases lung function, triggers exacerbations of asthma [4], and increases hospital admissions for asthma [5]–[7], especially for children [8]–[10]. Cold temperature is also one of major environmental factors that exacerbate chronic inflammatory airway diseases (for example, chronic obstructive pulmonary disease and asthma) [11]. Studies have shown that weather conditions play an important role in asthma attacks [12]. For example, there is a seasonal pattern in asthma admissions, with more during the wet season than the dry season in Mexico city [13]. The emergency room visitors for asthma in Oulu, Finland are higher in winter than summer [14]. For the short-term (day-to-day) effect of temperature, cold temperatures are related to acute exacerbations of asthma symptoms, while hot temperatures are associated with increased asthma prevalence which might be related to higher levels of allergen exposure [15], [16]. Some weather conditions like extremely hot or cold temperatures, changes in barometric pressure or humidity and wind can trigger asthma [17]–[19]. However, there is little data available on the effects of extreme cold temperatures on childhood asthma. This study investigated the association between cold spells and pediatric outpatient visits for asthma in Shanghai, China.

## Materials and Methods

### Study population

Shanghai is located on the east tip of Yangtze River Delta and along China's eastern coastline, at latitude 31° 14′ N and longitude 121° 29′ E. Shanghai covers an area of 6,341 square kilometers. Shanghai is the largest city by population in China, with a total population of over 23 million in 2010 including 1.99 million children (0–14 years) [20]. Weather in Shanghai is generally mild and moist, with four distinct seasons: a warm spring, a hot rainy summer, a cool autumn and a cold winter. The hottest time in Shanghai is usually between July and August while the coldest time is from the late January to early February.

### Data on pediatric outpatient visitors for asthma

We collected the retrospective data on daily counts of pediatric outpatient visits for asthma between 1 January 2007 and 31 December 2009 from the Shanghai Children's Medical Center (SCMC) affiliated to Shanghai Jiao Tong University School of Medicine. The SCMC is one of the largest pediatric research institutes in China. The primary diagnoses of daily outpatients were coded according to the International Classification of Disease, 9th revision for asthma (ICD9: code 493).

### Data on air pollution and weather conditions

Data on daily mean temperature, relative humidity and ozone (O_3_), were obtained from the Shanghai Meteorological Bureau. Daily mean temperature, relative humidity and O_3_ were calculated using the records from monitors throughout the urban area of Shanghai.

### Data analysis

#### 1. Cold spell definition

There is no standard definition of a cold spell worldwide [21]. Many methods were used to define cold spells [21]–[23]. For example, Huynen et al. defined a cold spell by using a period of at least 9 days with a minimum temperature of −5°C or lower [23]. We analysed several possible definitions of a cold spell, such as two or more consecutive days with temperature below the 1st, 2.5th or 5^th^ percentile of the temperature distribution. Finally, we defined a cold spell as four or more consecutive days with mean temperature below the 5^th^ percentile of the distribution during 2007–2009 as sensitivity analyses suggested it to be an appropriate cold spell definition for Shanghai (results not shown).

We used mean temperature (not minimum or maximum temperature) to define a cold spell, because it gave a best model fit as judged by quasi-Poisson Akaike Information Criterion (Q-AIC). The other reason is that mean temperature represents the exposure throughout the whole day, while minimum or maximum temperature only reflects the exposure for a short period. So mean temperature can be more easily interpreted for decision making purposes [24].

#### 2. Temperature impacts on pediatric outpatient visits for asthma

Both cold and hot temperatures increase the risk of morbidity, and the temperature-morbidity relationships are generally U-, V-, and J- shaped [25], [26]. Therefore, we only used data in cold season (from December to April) to explore the effect of temperature on pediatric outpatient visits for asthma. We used a time series model to explore the relationship between current day's temperature (continuous variable) and pediatric outpatient visits for asthma in cold seasons. We assumed that the daily number of outpatient visits was over-dispersed by quasi-Poisson function. As previous studies have shown that the temperature impact on morbidity was non-linear [25], [26], we used a spline with 3 degrees of freedom for temperature. We controlled for relative humidity and O_3_ using spline with 3 degrees of freedom. We controlled for day of the week as a category variable. We used a spline with 3 degrees of freedom for calendar day to control for season and a long-term trend. We plotted the relationship between temperature and pediatric outpatient visits for asthma.

#### 3. The effect of cold spells on pediatric outpatient visits for asthma

We used a time series model to estimate relative risks (RRs) (with 95% confidence intervals (CIs)) of pediatric outpatient visits for asthma by comparing the number of the visits during the cold spells with those during non-cold spells in cold seasons. Mean temperature was used to derive a dummy variable to categorise cold spell or non-cold spell days. We controlled for a similar range of confounding factors as indicated above.

#### 4. The effect of an intense cold spell on pediatric outpatient visits for asthma in 2008

Our preliminary analysis showed that there was an intense and long cold spell (20 days) in 2008. Thus, to make the best use of a good opportunity of this natural experiment, we examined the relation between exposure to this cold spell and pediatric outpatient visits for asthma through comparing its RRs with those during the same periods in 2007 and 2009, after adjustment for a similar range of confounding factors as indicated above. We did not select the reference period immediately after or before this cold spell, as studies have found that the cold spell-related morbidity (or mortality) is usually followed by temporary reduction in morbidity (or mortality) in subsequent weeks. The demographic characteristics were unlikely to change substantially in the neighbouring years. Therefore we selected the same period of the cold spell in the neighbouring winters as the reference. This method has been widely used to examine the effects of heat waves and cold spells on morbidity and mortality in previous studies [22], [27].

#### 5. Lag effects of cold spells

Many studies have shown that the effects of cold temperature on morbidity and mortality last more than weeks [25], [26]. So it is necessary to examine the lag effects of cold spells on pediatric outpatient visits for asthma. We examined the lag effects (lag 0, lag 1–2, lag 3–6, lag 7–14, lag 15–30, and lag 0–30) of cold spells on pediatric outpatient visits for asthma in this study [28].

Sensitivity analyses were performed through changing degrees of freedom for calendar day, relative humidity and O_3_. We also used different definitions for a cold spell. Values of *P*<0.05 (two-sided) were considered statistically significant. The R software (version 2.10.1, R Development Core Team 2009) was used to fit all models. The “mgcv” package was used to fit quasi-Poisson regression [29].

## Results

**Figure 1:**
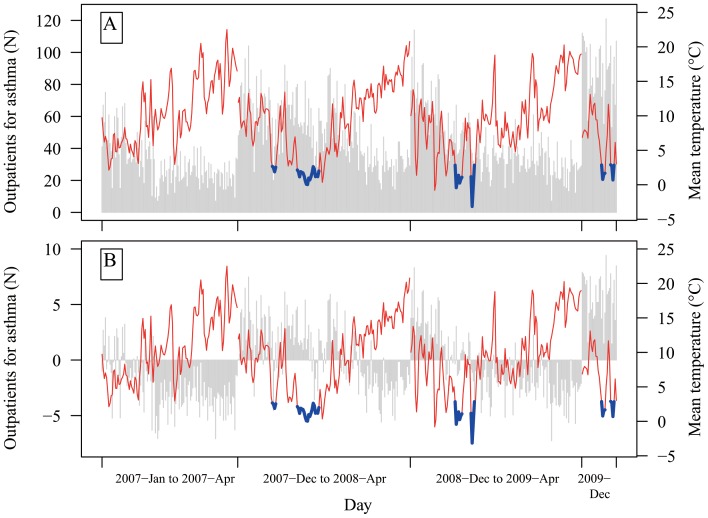
Time series of daily temperature and pediatric outpatient visits for asthma in cold seasons during 2007 and 2009 before (A) and after removing trends using a spline with 3 degrees of freedom for calendar time (B). The vertical bars are pediatric outpatient visits for asthma. The red lines are temperatures in non-cold spell days. The blue lines are temperatures in cold spell days.

There were 6 cold spells during 2007–2009 (Figure 1). Generally, in the extreme cold periods (not only cold spells), there were more paediatric outpatient visits for asthma than other periods (Figure 1).

Table 1 illustrates that during the 6 cold spells, the average temperature, relative humidity and O_3_ were 1.4°C, 62.4%, and 13.3 ug/m^3^, respectively, and there were 53 average (standard deviation: 19) pediatric outpatient visits for asthma. By contrast, in the non-cold spell days, the mean temperature, relative humidity and O_3_ were 10.3°C, 70.5%, 20.6 ug/m^3^, respectively, and there were 43 average (standard deviation: 22) pediatric outpatient visits for asthma. During the 20 days' cold spell in 2008, the number of pediatric outpatient visits for asthma was remarkably higher and mean temperature was significantly lower than that during the same periods in 2007 and 2009 (Table 1).

**Table 1:** Statistic summary for weather conditions, air pollution and pediatric outpatient visits for asthma during the cold spell and non-cold spell periods.

Period	Mean (Minimum, Maximum)			
	Mean temperature	Relative humidity	O_3_	Asthma outpatients
All Cold spells	1.4 (−3.2, 2.9)	62.4 (29.5, 95)	13.3 (4.8, 33.9)	53 (20, 104)
Non-cold spells during cold season	10.3 (−0.8, 22.5)	70.5 (34.5, 96.8)	20.6 (0.6, 59.5)	43 (7, 121)
20 days cold spell in 2008	1.4 (0, 2.6)	75.8 (59, 95)	16.1 (4.8, 33.9)	53 (24, 82)
The same period in 2007	7.3 (3.1,14.9)	65.4 (48.0, 91.3)	12.1 (6.1, 20.5)	38 (18, 68)
The same period in 2009	6.8 (−3.2, 12.3)	73.3 (21.5, 91.8)	14.4 (3.3, 35.4)	38 (19, 62)

**Figure 2:**
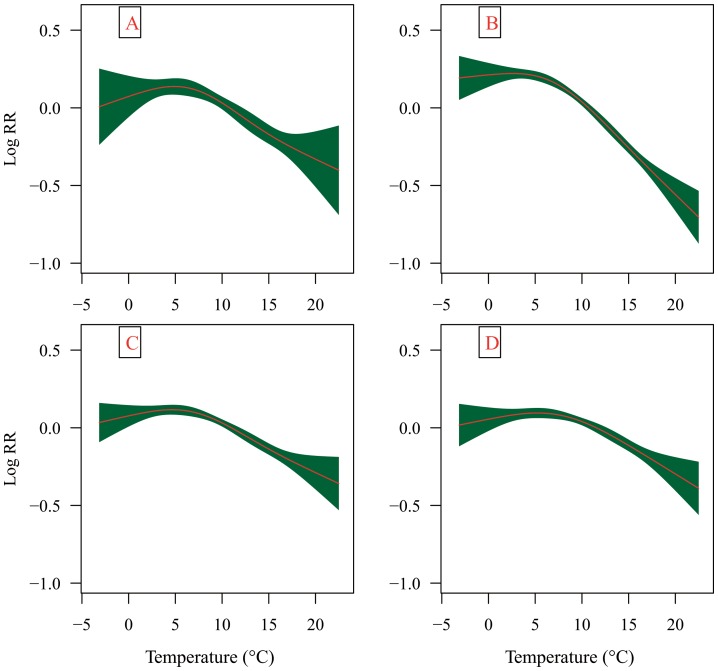
The relationship between temperature and pediatric outpatient visits for asthma in cold seasons during 2007 and 2009 after adjustment for trends and day of the week (A); trends, day of the week and relative humidity (B); trends, day of the week and O_3_ (C); trends, day of the week, relative humidity and O_3_ (D).

Figure 2 shows the relationship between current day's temperature and pediatric outpatient visits for asthma in cold seasons during 2007 and 2009. There were apparent cold effects on pediatric outpatient visits for asthma. In general, the lower the mean temperature, the higher the risk of pediatric outpatient visits for asthma.

We examined the relative risk for children's asthma during cold spells compared with the non-cold spell days during 2007 and 2009 (Table 2). We found that broadly there was a significantly increased risk for children's asthma associated with cold spells across different lags, compared with the non-cold spell days. However, the impact of cold spells was generally attenuated and only significant at lag 7–14 days after adjustment for the effect of O_3_.

**Table 2:** The relative risks (RRs) for pediatric outpatient visits for asthma during cold spells compared with non-cold spell days in cold seasons, 2007–2009^a^.

Lags	RR (95% CI)			
	Model 1^b^	Model 2^c^	Model 3^d^	Model 4^e^
Lag 0	1.25 (1.08, 1.44)**	1.25 (1.08, 1.45)**	1.06 (0.93, 1.20)	1.05 (0.92, 1.20)
Lag 1–2	1.29 (1.13, 1.47)**	1.30 (1.13, 1.48)**	1.11 (0.98, 1.25)	1.09 (0.96, 1.23)
Lag 3–6	1.29 (1.14, 1.46)**	1.29 (1.14, 1.47)**	1.09 (0.97, 1.22)	1.07 (0.95, 1.20)
Lag 7–14	1.26 (1.12, 1.42)**	1.26 (1.13, 1.42)**	1.12 (1.02, 1.25)*	1.12 (1.02,1.25)*
Lag 15–30	1.06 (0.95, 1.19)**	1.06 (0.95, 1.19)**	1.02 (0.92, 1.13)	1.03 (0.93, 1.14)
Lag 1–30	1.26 (1.14, 1.38)**	1.25 (1.14, 1.39)**	1.08 (0.98, 1.18)	1.06 (0.97, 1.17)

a All the models were adjust for day of the week, season and long-term trend;

b Model 1 did not controlled for relative humidity and O_3_;

c Model 2 controlled for relative humidity;

d Model 3 controlled for O_3_;

e Model 4 controlled for relative humidity and O_3_;

* <0.05;

** *P*<0.01.

We also examined the relative risk for children's asthma during the 20-day cold spell in 2008 compared with the same periods in 2007 and 2009 (Table 3). Results show that the 20-day cold spell significantly increased the risk for children's asthma at different lags, even after adjustment for confounding factors. The severe cold spell also seemed to exhibit a quite long lagged effect on the onset of children's asthma.

**Table 3:** The relative risk for pediatric outpatient visits for asthma during 20-day cold spell in 2008 compared with the same periods in 2007 and 2009^a^.

Lag	RR (95% CI)			
	Model 1^b^	Model 2^c^	Model 3^d^	Model 4^e^
Lag 0	1.40 (1.21, 1.61)	1.37 (1.18, 1.58)	1.45 (1.26, 1.67)	1.41 (1.22, 1.63)
Lag 1–2	1.44 (1.25, 1.66)	1.42 (1.22, 1.64)	1.51 (1.30, 1.75)	1.46 (1.25, 1.72)
Lag 3–6	1.47 (1.29, 1.68)	1.46 (1.27, 1.67)	1.49 (1.30, 1.71)	1.47 (1.27, 1.72)
Lag 7–14	1.71 (1.46, 1.99)	1.66 (1.41, 1.96)	1.75 (1.49, 2.06)	1.70 (1.43, 2.02)
Lag 15–30	1.68 (1.50, 1.93)	1.71 (1.43, 2.03)	1.72 (1.48, 2.00)	1.65 (1.36, 2.00)
Lag 1–30	1.62 (1.46, 1.79)	1.63 (1.46, 1.82)	1.65 (1.48, 1.84)	1.64 (1.46, 1.84)

a All the models were adjusted for day of the week, season and long-term trend; and all RRs were statistically significant (*P*<0.01);

b Model 1 did not controlled for relative humidity and O_3_;

c Model 2 controlled for relative humidity;

d Model 3 controlled for O_3_;

e Model 4 controlled for relative humidity and O_3_;

### Sensitivity analyses

Sensitivity analyses were conducted by changing the *df* of the splines for temperature, relative humidity and O_3_ and the *df* of smoothing for time. The results remained broadly similar (results not shown).

When different definitions for a cold spell were used, we found there were similar estimated effects of cold spells on pediatric outpatient visits for asthma. However, the definition of four or more consecutive days with temperature below the 5^th^ percentile of temperature gave a best model fit as judged by quasi-Poisson Akaike information criterion.

## Discussion

A number of studies have assessed the climate impact on respiratory mortality [30], but relatively few studies have examined the relationship between extreme cold temperatures and respiratory morbidity [25], [26]. In addition, no identified study has investigated the association between cold spells and pediatric outpatient visitors for asthma. According to our knowledge, this is the first study to investigate the association between cold spells and pediatric outpatient visits for asthma in Shanghai, China. In 2008, there was a severe cold spell in winter in Shanghai, which provided a good opportunity to examine the cold effect on asthma morbidity.

In this study, we found that cold temperature may significantly increase the risk of the outpatient for children's asthma. We assessed the relative risk for children's asthma during the 20-day cold spell in 2008 compared with the same periods in 2007 and 2009. Results show that the 20-day cold spell remarkably increased the risk of pediatric outpatient visitors for asthma. We also examined the relative risks of pediatric outpatient visitors for asthma during cold spells compared with non-cold spell days in cold seasons during 2007 and 2009. There was still a significant impact on the utilisation of health services for asthma but this impact was alleviated after controlling for the confounding effects of O_3_. These findings suggest that extreme cold temperature may trigger asthma.

Some epidemiological studies have found that cold temperature is associated with respiratory morbidity and mortality [25], [26]. Cold temperature is related to an increased risk of significant exacerbation of asthma [19]. Cold temperature–related exacerbation of those diseases is often followed by a subsequent increase in bacterial and viral infections of the airway, infiltration of inflammatory factors, and mucus secretion [11], [31], [32]. Cold temperature is associated with increased occurrence of respiratory tract infections, and a decrease in temperature often proceed the onset of the infections [33].

Study shows that cold temperature induces mucin hypersecretion from normal human bronchial epithelial cells *in vitro* through a transient receptor potential melastatin 8 [11]. A radionuclide perfusion study shows that rabbits exposed to cold temperature had lesser lung perfusion than controls. Cold temperature can induce contraction of the trachea and decrease pulmonary circulation and lung perfusion. This summation of acute cooling for tracheal smooth muscle and pulmonary circulation might be the reason why severe cold temperature induces contraction [34].

When examining the effects of cold spells on pediatric outpatients for asthma, we found that the cold effects were attenuated after controlling for O_3_ effects. However, when we only assessed the risk for children's asthma during the 20-day cold spell in 2008 compared with the same periods in 2007 and 2009, the cold effects were still significant, even after controlling for O_3_. The reason might be because there is an interactive effect of O_3_ and temperature on respiratory health [35]. In cold seasons, the O_3_ concentration was higher in non-cold spell days than cold spell days (Table 2). This situation will affect the assessment of the effects of cold spells on the risk for children's asthma. By contrast, the O_3_ concentration in the 20-day cold spell was similar as the same periods in 2007 and 2009 (Table 2). The impact of temperature in those periods was unlikely to be modified by O_3_.

This study found that cold spells had a long lagged effect on children asthma. Although no data is available on the relationship between cold spells and children asthma, several studies have reported that cold temperatures had a relatively long lagged effect on mortality and morbidity (including non-external, cardiovascular, and respiratory) [25], [36], [37]. These findings suggest that the lagged effects of cold spells are important and should not be overlooked. When healthcare providers and public health authorities develop response plans to protect children with asthma from cold temperatures, it is necessary to consider the delayed impact of cold spells on asthma exacerbations.

In this study, we used Poisson regression model to examine the effect of cold spells on pediatric outpatient visits for asthma. Alternatively, case-crossover design can also be used to examine the short-term effect of temperature (or air pollution) on mortality and morbidity. Previous studies have compared these two designs, and found that they are comparable and produce similar effect estimates [38], [39]. To make sure our results are reliable, we used a time-stratified case-crossover analysis to examine the relationship between cold spells and pediatric outpatient visits for asthma. The results are similar as those from Poisson regression model.

We used multiple comparisons to examine the effects of cold spells on pediatric outpatient visits for asthma. All these analyses have shown that cold spells were associated with the increased pediatric outpatient visits for asthma. The multiple comparisons may produce spurious statistical associations because a large dataset was used. However, our sensitivity analysis using case-crossover study also gave the similar results (results not shown).

We are aware that well controlled patients do not necessarily attend a hospital clinic, and may see a general practitioner, change beheviour (i.e. won't go outside) or take medications. It means that the effects of cold temperatures on pediatric outpatient visits for asthma might be under-estimated. Additionally, even though a clear and consistent relation between cold spells and pediatric outpatient visitors for asthma was observed, we cannot conclude that the increase in child asthmatic attacks was directly caused by cold spells. There are some confounding factors that we are unable to control for. For example, is asthma exacerbation caused by the cold weather *per se* or triggered by viral infections during cold weather? Because data on viral infections were not available, we could not adjust for its influence. Therefore, the mechanisms underlying the relationship between cold spells and pediatric asthmatic attacks should be examined in future research.

### Strengths and Limitations

According to the best of our knowledge, this is the first study to examine the impact of cold spells on children's asthma. We used the opportunity of a natural experiment that there was a long cold spell in Shanghai in 2008. We found that cold temperatures, particularly severe cold spells, significantly increased the risk of the onset of children's asthma. This evidence is useful for controlling and preventing children's asthma in cold seasons, especially in developing countries. Secondly, we examined the lagged effects of cold spells on children's asthma in detail and found that extreme cold temperatures had quite long delayed effects on the onset of children's asthma.

This study also has some limitations. We only used the data on pediatric outpatient visits for asthma from one children's hospital in one city. Even though we controlled for the effect of O_3_, other air pollutants were not adjusted for in the model (the data on other air pollutants were incomplete for the study period). However, previous studies showed that temperature effects on health outcomes are generally robust and independent to air pollution [37], [40], [41]. This is an ecological study. Individual exposure data was not available, and thus measurement bias is inevitable to some extent. The cold spell definition used in this study is difficult to be generalised to other countries, especially for higher latitude regions, because people can adapt to their local climate.

### Conclusion

Cold temperatures significantly increased the risk of pediatric outpatient visits for asthma. The findings suggest that extreme temperature events such as cold spells may trigger asthmatic attacks. Children with asthma need to be well protected from cold effects in winter.
